# Modeling the relationship between precipitation and malaria incidence in Mpumalanga, South Africa

**DOI:** 10.1186/1475-2875-11-S1-P127

**Published:** 2012-10-15

**Authors:** Sheetal P Silal

**Affiliations:** 1 Department of Statistical Sciences, University of Cape Town, Cape Town 7700, South Africa

## Background

Climatic or weather-driven factors such as rainfall have considerable impact on vector abundance and the extrinsic cycles that parasites undergo in mosquitoes [1]. Climate models therefore allow for a better understanding of the dynamics of malaria transmission [2]. While malaria seasons occur regularly between October and May in Mpumalanga, there is considerable variation in the starting point, peak and magnitude of the season. The relationship between rainfall and malaria incidence may be used to better model the variation in the malaria season. As a first step, this study seeks to explore the complex association between rainfall and malaria incidence through time series methods.

## Materials and methods

The statistical relationships between weekly malaria case data and accumulated weekly rainfall were explored for Mpumalanga in the period 2002 and 2010. Two analyses were performed; namely, cross correlations of the raw data series and cross correlations of the pre-whitened data series. Pre-whitening is achieved by using the Box Jenkins approach to fit Seasonal Autoregressive Integrated Moving Average (SARIMA) models.

## Results

Cross correlation analysis of the raw rainfall and case series yielded significant negative correlations between lag -20 and lag -40 and significant positive correlations between lags -4 and -10. The cross correlations analysis of the transformed rainfall and case series (with stabilized variance) yielded significant negative correlations between lag -40 and lag -18 and significant positive correlations between lag -13 and lag 2. The analysis of the pre-whitened series showed that many of these correlations were spurious. A SARIMA (1,1,2)(0,1,2)_52_ model was fitted to the transformed rainfall series and applied to the transformed case series and a cross correlation analysis of the residuals of these two SARIMA models showed significant positive correlations at lags -5 and -6.

## Conclusions

The relationship between rainfall and malaria incidence is non-direct and complex. The consequence of pre-whitening is to reduce unassociated autocorrelations in the time series before the cross correlations are computed, thereby reducing the number of spurious correlations. Lagged rainfall data has the potential to be used in place of trigonometric functions to model the variable seasonality component in mathematical models of malaria transmission.
